# Whole-Genome Sequencing of Two Potentially Allelopathic Strains of *Bacillus* from the Roots of *C. equisetifolia* and Identification of Genes Related to Synthesis of Secondary Metabolites

**DOI:** 10.3390/microorganisms12061247

**Published:** 2024-06-20

**Authors:** Ying Wang, Pan Chen, Qi Lin, Linzhi Zuo, Lei Li

**Affiliations:** Ministry of Education Key Laboratory for Ecology of Tropical Islands, Key Laboratory of Tropical Animal and Plant Ecology of Hainan Province, College of Life Sciences, Hainan Normal University, Haikou 571158, China

**Keywords:** *Bacillus amyloliquefaciens*, *Bacillus aryabhattai*, allelopathy, whole-genome sequencing, *Casuarina equisetifolia*, allelochemical synthesis-associated genes

## Abstract

The coastal *Casuarina equisetifolia* is the most common tree species in Hainan’s coastal protection forests. Sequencing the genomes of its allelopathic endophytes can allow the protective effects of these bacteria to be effectively implemented in protected forests. The goal of this study was to sequence the whole genomes of the endophytes *Bacillus amyloliquefaciens* and *Bacillus aryabhattai isolated* from *C. equisetifolia* root tissues. The results showed that the genome sizes of *B. amyloliquefaciens* and *B. aryabhattai* were 3.854 Mb and 5.508 Mb, respectively. The two strains shared 2514 common gene families while having 1055 and 2406 distinct gene families, respectively. The two strains had 283 and 298 allelochemical synthesis-associated genes, respectively, 255 of which were shared by both strains and 28 and 43 of which were unique to each strain, respectively. The genes were putatively involved in 11 functional pathways, including secondary metabolite biosynthesis, terpene carbon skeleton biosynthesis, biosynthesis of ubiquinone and other terpene quinones, tropane/piperidine and piperidine alkaloids biosynthesis, and phenylpropanoid biosynthesis. NQO1 and entC are known to be involved in the biosynthesis of ubiquinone and other terpenoid quinones, and rfbC/rmlC, rfbA/rmlA/rffH, and rfbB/rmlB/rffG are involved in the biosynthesis of polyketide glycan units. Among the *B. aryabhattai*-specific allelochemical synthesis-related genes, STE24 is involved in terpene carbon skeleton production, atzF and gdhA in arginine biosynthesis, and TYR in isoquinoline alkaloid biosynthesis. *B. amyloliquefaciens* and *B. aryabhattai* share the genes aspB, yhdR, trpA, trpB, and GGPS, which are known to be involved in the synthesis of carotenoids, indole, momilactones, and other allelochemicals. Additionally, these bacteria are involved in allelochemical synthesis via routes such as polyketide sugar unit biosynthesis and isoquinoline alkaloid biosynthesis. This study sheds light on the genetic basis of allelopathy in Bacillus strains associated with C. equisetifolia, highlighting the possible use of these bacteria in sustainable agricultural strategies for weed management and crop protection.

## 1. Introduction

The coastal *Casuarina equisetifolia* is currently a significant coastal tree in southeastern China and plays an essential part in protecting the coastal ecosystem [1,2]. However, as *C. equisetifolia* hit the second-generation update stage, there were evident serialization issues. It faces a number of issues, including a loss in planted forest soil strength, difficulty in regeneration, and a decline in production, all of which have major ramifications for the coastal ecosystem [3]. Previous research has indicated that one of the primary causes of *C. equisetifolia* plantation loss is the continual synthesis and accumulation of allelochemicals [4]. *C. equisetifolia* has abundant microorganisms, which promote plant growth and increase disease and pest resistance [5,6], as well as facilitating plant capacity for salt and drought resistance, all of which are important in ecological restoration and resistance to natural disasters in coastal regions [7,8]. The ecological systems driving allelopathy and within-plant microorganism transmission are complex and poorly understood. Allelopathy is the term used to describe the positive or negative effects that microbes or plants can have on other plants by releasing chemicals into their surroundings, either directly or indirectly, and it is essential to agricultural activities and ecological interactions [9]. Allelochemicals are the carriers used for transferring allelopathic information and signals. Numerous processes, including root secretion, leaching through rain and fog, spontaneous evaporation, and plant litter decomposition, release these chemicals into the surrounding environment [10]. Moreover, they continuously accumulate in plants and the surrounding environment, thereby controlling plant growth and development. Allelochemicals are almost always secondary metabolites of plants or microorganisms and primarily include organic acids, ketones, terpenoids, phenols, alkaloids, glycosides, amino acids, and peptides [11,12]. Numerous studies suggest that plant endophytes can create secondary metabolites that are similar or identical to those of the host plant, thereby boosting the host plant’s allelopathy [13,14]. The production of these secondary metabolites may derive from the decomposition of litter or from litter endophytes and microorganisms adhering to litter surfaces. Recent studies have emphasized the potential for specific Bacillus strains to exhibit allelopathic traits, thereby providing interesting options for natural pesticide development and sustainable farming practices.

Plants actively seek the assistance of microorganisms to survive in stressful environments. Over the last decade, plant research has undergone a paradigm change, with the discovery of microbial roles and community composition increasingly viewed as drivers for improving plant host performance [15]. The endophytic microbiome has the ability to increase plant genomes and metabolic capacities, allowing it to supply or encourage a variety of tasks that assist plants in maintaining essential life activities, such as nutrition acquisition, immunological control, and biological stress tolerance. During the past few decades, scientists have worked to understand why and how plants and microbes interact differently in their environments. One of the main factors responsible for such behavior is the genotype, which includes instructions for adaptation and survival [16]. The rhizosphere is a thin film of soil that surrounds plant roots and is the major site of nutrient intake. It also hosts significant physiological, chemical, and biological activities. Many bacteria penetrate the rhizosphere and can boost plant growth and health [17]. Species of Bacillus are Gram-positive sporeformers, and many of these species are known for their adaptable metabolic capacities and profound effects on soil ecosystems and plant health [18]. In a recent study, it was shown that Bacillus–plant interactions result in improved plant development and defenses because they are stimulated by chemical communication involving an array of metabolites [19,20]. In our previous investigation, which involved the isolation of the endophytes *Bacillus amyloliquefaciens* and *Bacillus aryabhattai* from the roots of *Casuarina equisetifolia*, we observed that these two strains exhibited considerable allelopathic potential in comparison to other endophytes [21]. In addition, previous studies on these two strains have focused on their use in agricultural biofertilizers and biocontrol, which can induce systemic tolerance to abiotic stresses by triggering genetic, chemical, and physical changes in the host plant [22,23,24,25]. However, genomic information on these two strains, notably genes involved in biological pathways connected with allelochemical production, has yet to be identified.

When diverse microorganisms live in the same habitat, they have to fight with one another for the resources needed for reproduction, sustenance, and other necessities during their life cycles. Organisms have evolved two effective competitive tactics to ensure their survival. One is the production of allelochemicals that hinder the growth of their competitors and reduce detrimental effects caused by their competitors in the surrounding area [26,27,28]. The other is the production of allelochemicals that allow the makers to form alliances with symbionts or hosts. These mutually beneficial relationships allow both sides to develop successfully and reproduce securely, even in the most adverse environments [26]. Therefore, Bacillus secretes a variety of helpful secondary metabolites, and when isolated from various environments the bacterium has a high genetic diversity [29]. For example, in an in vitro competition trial, naphthoquinone spiroketals, isolated from a recently discovered EF strain, showed an allelochemical inhibitory impact against other EFs, such as *Colletotrichum* sp. and *Phomopsis* sp. [30]. In another in vitro dual-culture experiment, it was discovered that *P. variabile*, an EF, exhibited direct antagonism against *F. oxysporum*, a phytopathogen, by secreting the induced metabolite hydroperoxin oxylipin, which reduced the concentration of pathogenic mycotoxins, whereas none of the pure axenic cultures showed any increase or decrease in this metabolite [31]. Further research has found that lipoxygenases and the two lipoxygenase genes (pvlox1 and pvlox2) in *P. variabile* catalyze the manufacture of hydroperoxy oxylipins, with only pvlox2 specifically up-regulated during the contact [32]. Further study showed that primary metabolites are the byproducts of primary metabolic pathways, such as carbohydrates, amino acids, proteins, and lipids. They play an important metabolic role in the formation and development of an organism. Without them, the organism’s growth and development are highly susceptible to faults. Primary metabolism plays a crucial role in supplying precursors for the production of SMs. Endophytes and host plants use these precursors in their respective SM biosynthesis processes. The biosynthetic pathway of SMs in EFs could be the result of their copying the host pathways [33,34]. Despite important studies on plant allelopathy, the particular contributions of rhizosphere-associated Bacillus strains to the allelopathic effects observed in *C. equisetifolia* are mainly unknown.

NGS has enhanced research in transcriptomics, epigenomics, metagenomics, and other omics domains in addition to complete genome sequencing [35,36,37,38]. Whole-genome sequencing (WGS) offers a deep understanding of organisms’ genetic blueprints, allowing the discovery of genes involved in allelopathic activity while providing insights into their molecular and functional mechanisms [39]. In this paper, we report the whole-genome sequences of two Bacillus strains (*B. amyloliquefaciens* and *B. aryabhattai*) from the roots of *C. equisetifolia* suspected of allelopathic activity. We used a combination of Illumina Hiseq and single-molecule PacBio sequencing technologies to obtain genome-wide information and to screen the strains’ genomes for genes associated with allelochemical synthesis. Our goals were, first, to give thorough genomic profiles of the two Bacillus strains and then to identify and annotate genes involved in the manufacture of secondary metabolites that may contribute to allelopathic interactions. The results of this research will shed light on the complex interplay between *C. equisetifolia* and its rhizosphere microbiome, contributing to our understanding of plant–microbe interactions and allelopathic ecological dynamics.

## 2. Materials and Methods

### 2.1. Isolation of B. amyloliquefaciens and B. aryabhattai

The test strains were *B. amyloliquefaciens* and *B. aryabhattai*, endophytes previously isolated from *C. equisetifolia* roots. The strains were identified using colony morphology, Gram staining, and species identification [21]. The *B. amyloliquefaciens* strain was designated XYG6, whereas *B. aryabhattai* was designated as XAG3.

Regarding bacterial culture and preparation, the endophytes *B. amyloliquefaciens* XYG6 and *B. aryabhattai* XAG3 were identified using criteria based on the colony and were purified on LB solid media. Single colonies were picked and inoculated into an LB liquid medium. They were cultured at 37 °C, incubated at 170 r/min overnight, and inoculated into a new LB liquid medium the following day at a 1% ratio. These were cultured to the logarithmic growth phase and centrifuged at 170 r/min at 37 °C. An appropriate volume of bacterial suspension was transferred to a centrifuge tube and centrifuged at 14,000× *g* for 5 min at 25 °C. The supernatant was discarded and the precipitate was transferred to a 1.5 mL sterile freezing tube, and 1.5–2.0 g of the bacterial precipitate was weighed.

### 2.2. Genomic DNA Extraction, Whole-Genome Sequencing, and Sequence Assembly

XYG6 and XAG3 genomic DNA was extracted using a bacterial genomic DNA extraction kit (Tiangen Biotechnology Co., Ltd., Beijing, China). Whole-genome sequencing of the bacteria was performed using a combination of Illumina Hiseq and single-molecule PacBio sequencing technologies. The Illumina Hiseq sequencing process included genomic DNA purification, library construction, bridge PCR, and sequencing. The single-molecule PacBio sequencing process included genomic DNA purification and detection, genomic DNA fragmentation (g-TUBE method), library construction, sequencing, and quality assessment, which resulted in 99% base accuracy. De novo assembly of the obtained sequencing data was performed. Bacterial genome scans were assembled from optimized sequences obtained from next-generation sequencing with multiple Kmer parameters, using the short sequence assembler SOAPdenovo2 (Version 2.04) to obtain optimal contig assembly results. Next, the reads were aligned with the contigs, and local assembly and optimization of the assembly results were performed based on the paired-end and overlap relationships of the reads. Hybrid genome assembly of complete bacterial genome maps was performed using the unicycler assembler [40]. Sequence correction during the assembly process was performed using Pilon software (Version 1.22) [41]. Finally, the complete chromosome and plasmid sequences were obtained.

### 2.3. XYG6 and XAG3 Gene Prediction and Functional Annotation

PlasFlow software (Version 1.1) was used for plasmid identification of the bacterial genome assembly results. BLAST software (Version 2.3.0) and the PLSDB database (https://ccb-microbe.cs.uni-saarland.de/plsdb/, accessed on 27 June 2022) were used for plasmid annotation of the obtained plasmid sequences. Coding sequences (CDSs) in the genomes were predicted using Glimmer (Version 3.02), GeneMarkS (Version 4.3), and Prodigal software (Version 2.6.3) [42,43]. Genome scan assembly results were predicted using Glimmer, and the complete map assembly results were predicted using Glimmer for chromosome genomes and GeneMarkS for plasmid genomes [43]. tRNAscan-SE (Version 2.0) was used for the prediction of tRNA genes in the genomes to obtain the nucleotide sequence, anticodon, and secondary structure information of tRNAs in the genome of each sample [44]. Barrnap (Version 0.8) was used to predict rRNA genes in the genome and obtain information on the type, location, and sequence of all rRNAs in the genome of each sample.

Two methods of functional annotation of the predicted coding genes were used: 1. gene function annotation by homology alignment with selected reference genomes; 2. functional annotation by alignment with six major databases (NR, Swiss-Prot, Pfam, EggNOG, GO, and KEGG). Gene annotation was based primarily on the alignment of protein sequences. The gene sequences were aligned using various databases to retrieve the corresponding functional annotation data. These two methods were also used in the screening and functional annotation of genes associated with allelochemical synthesis.

### 2.4. Comparative Analysis of the XYG6 and XAG3 Genomes

Based on genome mapping and sequencing data, homologous gene analysis using OrthoMCL allowed for gene family classification and an assessment of the number of specific genes and information corresponding to a particular gene family for each sample [45]. Phylogenetic trees were constructed based on 16S rDNA gene sequences. The original sequences were first aligned and corrected to obtain a matrix. Then, the best model for the matrix was evaluated using the jModelTest model prediction software (Version 2.1.10), which includes a variety of models, such as the Akaike information criterion (AIC) and the Bayesian information criterion (BIC), for the selection and evaluation of the models. The bootstrap method was selected to test the tree throughout the phylogenetic tree construction process.

## 3. Results

### 3.1. Characteristics of the XYG6 and XAG3 Genome Sequences

**Figure 1:**
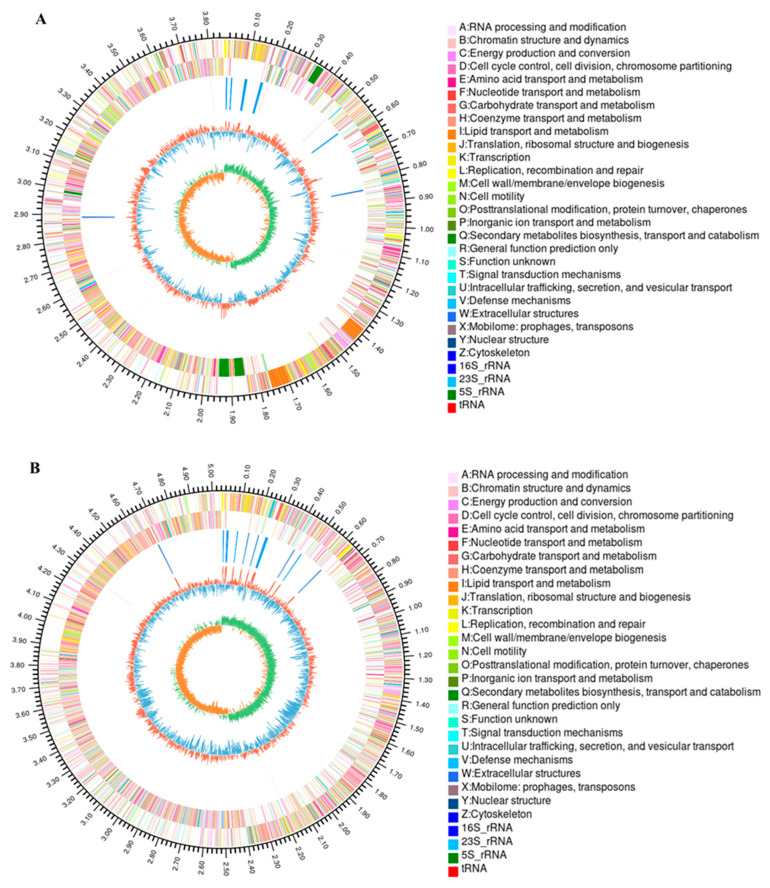
Genomic maps of XYG6 and XAG3. (**A**) Genomic map of XYG6. (**B**) Genomic map of XAG3.

The genomes of two *C. equisetifolia* endophytes, *B. amyloliquefaciens* (XYG6) and *B. aryabhattai* (XAG3), were sequenced using the Illumina Hiseq system. A total of 151,424 reads (base length: 1,414,508,947 bp) were collected for XYG6, with the smallest and longest sequences measuring 30 bp and 231,221 bp, respectively, and an average sequence length of 9341.38 bp. XAG3 yielded 268,897 reads (base length: 2,653,740,081 bp), with the smallest and longest sequences being 30 bp and 339,722 bp, respectively, and an average sequence length of 9868.98 bp. After assembly, the genome sizes of XYG6 and XAG3 were 3,854,088 bp and 5,507,526 bp and their GC contents were 46.37% and 38.07%, respectively. Circular genome maps for XYG6 and XAG3 were constructed using Circos (Figure 1A,B).

Our results showed that the XYG6 genome consisted of one chromosome, whereas the XAG3 genome consisted of one chromosome and seven plasmids. The XYG6 and XAG3 genomes contained 3744 and 5580 coding genes, respectively, and the length of the coding genes accounted for 88.56% and 82.81% of the respective total genome lengths. The XYG6 and XAG3 genomes contained 86 and 134 tRNA genes and 27 and 42 rRNA genes, respectively (Table 1).

### 3.2. Functional Annotation of the XYG6 and XAG3 Strains

**Figure 2:**
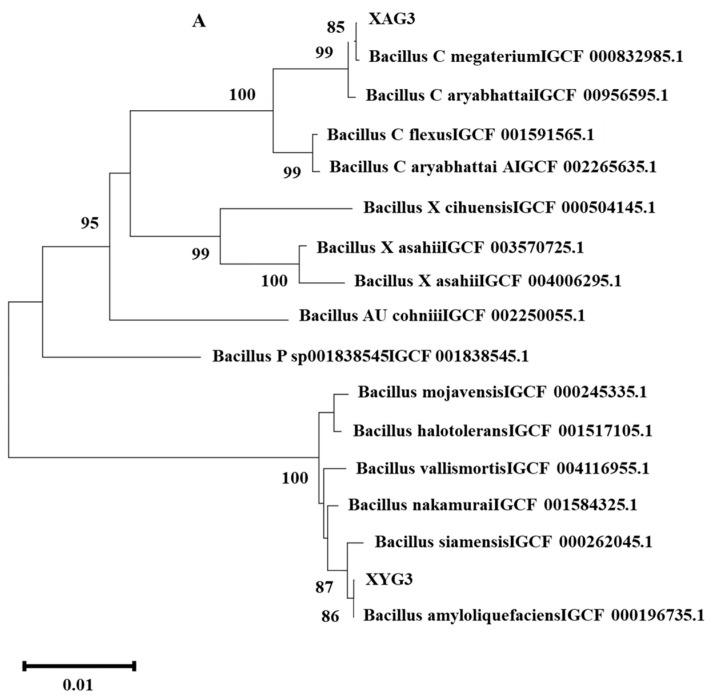

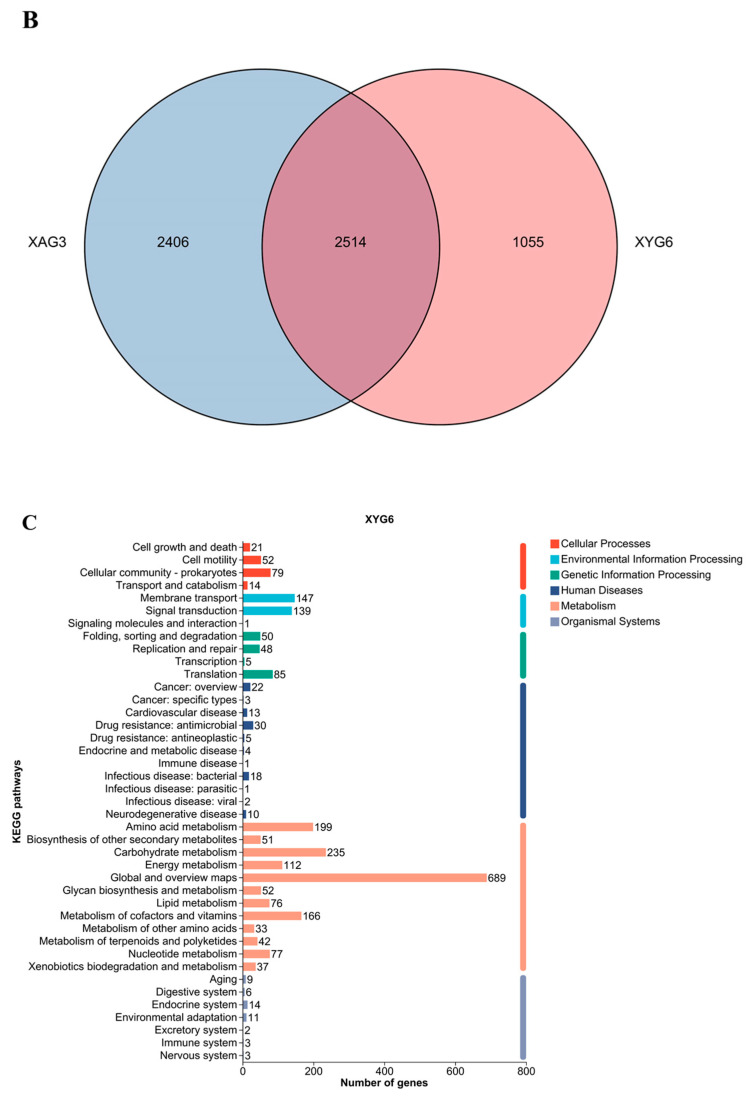

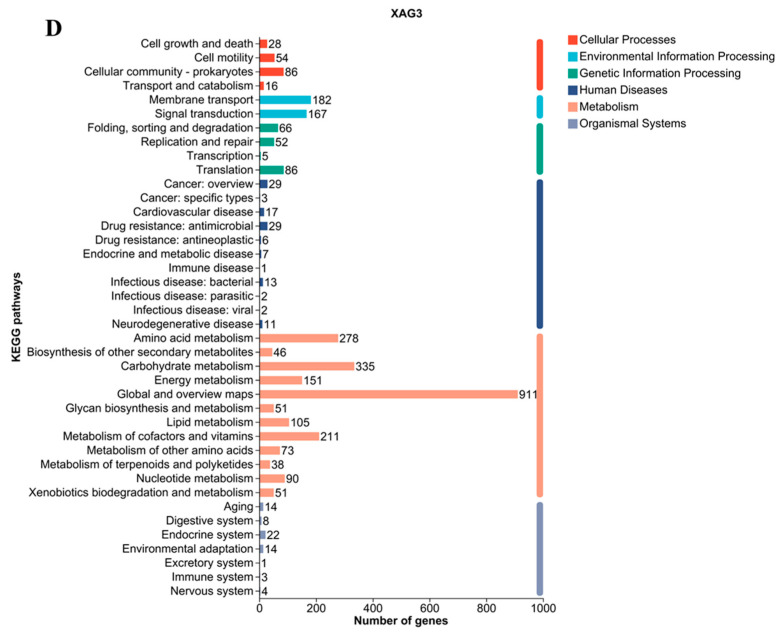
Functional annotation of the genes in the XYG6 and XAG3 strains. (**A**) Phylogenetic tree based on the 16S rDNA gene sequences. (**B**) Gene families are common and specific to XYG6 and XAG3. KEGG functional classification annotations for the XYG6 (**C**) and XAG3 (**D**) strains.

Phylogenetic tree analysis based on 16S sequences showed that XYG6 was closely related to *B. amyloliquefaciens* GCF 000196735.1 and that XAG3 was closely related to *Bacillus C megaterium* GCF 000832985.1 (Figure 2A). Homologous gene analysis revealed that XYG6 and XAG3 shared 2514 gene families and had 1055 and 2406 unique gene families, respectively (Figure 2B). Similarly, KEGG analysis revealed 2310 and 2930 gene annotations in the XYG6 and XAG3 strains, respectively. The annotations were clustered into six key categories: metabolism, organismal systems, human diseases, environmental information processing, cellular processes, and genetic information processing, with 41 functional pathways included. Numerous gene annotations were discovered in the metabolic and environmental information processing pathways. For the metabolic pathways, the number of genes annotated in XYG6 and XAG3 was 1769 and 2340, respectively, with 42 and 38 annotated genes related to the metabolism of terpenoids and polyketides, accounting for 2.37% and 1.62% of all genes annotated to the metabolic pathways. The number of genes annotated in the XYG6 and XAG3 genomes related to the biosynthesis of other secondary metabolites was 51 and 46, respectively, accounting for 2.88% and 1.96% of all genes annotated to the metabolic pathways, respectively (Figure 2C,D).

### 3.3. Allelochemical Synthesis-Related Genes in the XYG6 and XAG3 Genomes

**Figure 3:**
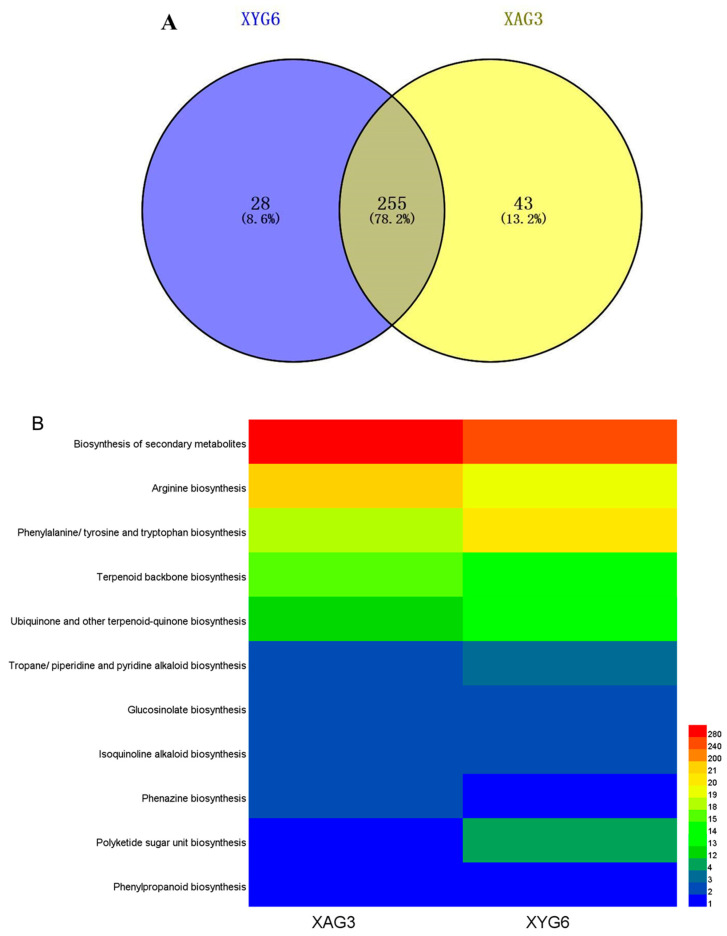
Allelochemical synthesis-related genes in the XYG6 and XAG3 genomes. (**A**) The number of allelochemical synthesis-related genes common and unique to the XYG6 and XAG3 genomes. (**B**) The number of allelochemical synthesis-related genes in the XYG6 and XAG3 genomes and their corresponding functional pathways.

Using the KEGG database for gene annotation, the number of genes associated with allelochemicals in the XYG6 and XAG3 genomes was 283 and 298, respectively. These comprised 255 genes related to the synthesis of allelochemicals shared between the two strains and 28 and 43 genes related to the synthesis of allelochemicals specific to each strain, respectively (Figure 3A). The genes were involved in 11 functional pathways, including secondary metabolite biosynthesis, terpene carbon skeleton biosynthesis, biosynthesis of ubiquinone and other terpene quinones, biosynthesis of tropane/piperidine and piperidine alkaloids, and phenylpropanoid biosynthesis (Figure 3B).

The allelochemical synthesis-related genes shared by XYG6 and XAG3 were primarily involved in secondary metabolite synthesis; terpene carbon skeleton biosynthesis; biosynthesis of ubiquinone and other terpene quinones; phenylpropanoid biosynthesis; phenylalanine, tyrosine, and tryptophan biosynthesis; arginine biosynthesis; phenazine biosynthesis; polyketide unit biosynthesis; glucosinolate biosynthesis; isoquinoline alkaloid biosynthesis; and tropan biosynthesis. In addition, the genes associated with secondary metabolite synthesis included *accA*, *ALDH*, *cysE*, *dapL*, *fabI*, *fbp3*, *gcvPA*, *hemA*, *hemQ*, *malZ*, *menB*, *pcrB*, *purC*, *purS*, *sirB*, *trpA*, *xpt*, and *yvoF*. The genes associated with terpene carbon skeleton biosynthesis were *ispD-F*, *ispH/lytB*, *ACAT/atoB*, *hepS-T*, *gcpE/ispG*, *dxr-s*, *GGPS*, *ubiX/bsdB/PAD1*, *uppS*, and *Idi/IDI.* The genes associated with the biosynthesis of ubiquinone and other terpene quinones were *wrbA*, *menA-F*, *menH*, *menI/DHNAT*, *ubiE*, and *ubiX/bsdB/PAD1.* The genes associated with phenylpropanoid biosynthesis were *ubiX/bsdB/PAD1.* The genes related to phenylalanine, tyrosine, and tryptophan biosynthesis were *aroK/aroL*, *aroG/aroA*, *hisC*, *tyrA2*, *trpA-F*, *aspB*, *aroA-C*, *aroE*, *pheB*, and *pheA2.* The genes associated with arginine biosynthesis were *gudB/rocG*, *rocF/arg*, *glsA/GLS*, *glnA/GLUL*, *purQ*, *OTC/argF/argI*, *argB-E*, *argJ*, *aspB*, *ureA-C*, *nos*, *argH/ASL*, and *argG/ASS1.* The gene associated with phenazine biosynthesis was *trpE*. The gene associated with polyketide unit biosynthesis was *rfbD/rmlD.* The genes associated with glucosinolate biosynthesis were *ilvE* and *leuC/IPMI-L*. The gene associated with isoquinoline alkaloid biosynthesis was *aspB.* The genes associated with tropane/piperidine and piperidine alkaloid biosynthesis were *aspB* and *yhdR*. Among the XYG6-specific allelochemical synthesis-related genes, *NQO1* and *entC* were involved in the biosynthesis of ubiquinone and other terpene quinones, and *rfbC/rmlC*, *rfbA/rmlA/rffH*, and *rfbB/rmlB/rffG* were involved in polyketide unit biosynthesis. In addition, the genes *adhP*, *crtB*, *entA*, *ghrB*, *purT*, *bacC*, and *yxeI* were involved in secondary metabolite biosynthesis. Among the XAG3-specific allelochemical synthesis-related genes, atzF and gdhA were involved in arginine biosynthesis, phzF was involved in phenazine biosynthesis, STE24 was involved in terpene carbon skeleton biosynthesis, and TYR was involved in isoquinoline alkaloid biosynthesis. In addition, the genes aarC/cat1, crtQ, FDFT1, gph, korA/oorA/oforA, and rpiA were involved in secondary metabolite biosynthesis.

## 4. Discussion

Genetics and edaphic factors are crucial for plant growth and productivity. Plants and other organisms like bacteria and fungi establish complex reciprocal relationships in most natural environments [46]. Numerous microorganisms associated with plants alter plant physiology and metabolism in addition to making plants more resilient to biotic and abiotic stressors [47]. These interactions can either promote or inhibit growth, which has a substantial impact on plant health. Integrating the plant microbiome to improve the management and modification of non-crop microbiomes is becoming a more widely accepted long-term strategy. As a result, understanding the ecological processes that govern microbiome assembly is critical.

In the current study, the genetic information of the allelopathic bacteria *B. amyloliquefaciens* XYG6 and *B. aryabhattai* XAG3 was obtained using whole-genome sequencing. The results showed that *B. amyloliquefaciens* (XYG6) had a genome size of 3.854 Mb and a GC content of 46.37% and that *B. aryabhattai* (XAG3) had a genome size of 5.508 Mb and a chromosome sequence length of 5.040 Mb, with GC contents of 38.07% and 38.36%, respectively. Similarly, the genome size of *B. amyloliquefaciens* subspecies *Lactiplantibacillus plantarum* strain (B9601-Y2), a plant growth-promoting bacterium isolated from the wheat rhizosphere, was found to be 4.243 Mb, with a GC content of 45.85% [48]. Similar to our work, a previous study indicated that the genome size of *B. amyloliquefaciens* (KNU-28) isolated from peach leaves was 4.239 Mb, with a GC content of 45.9% [49]. In addition, the genome size of *B. amyloliquefaciens* (L-S60), a plant growth-promoting bacterium isolated from the soil, was found to be 3.903 Mb, with a GC content of 46.67% [50]. The genome size of *B. amyloliquefaciens* (UASWS BA1), an antifungal strain isolated from sycamore tree tissues, was found to be 3.944 Mb, with a GC content of 46.6%. Consistent with our results, previous research revealed that plant growth-promoting *B. amyloliquefaciens* includes subspecies with highly varied whole-genome sequence sizes, ranging from 3.86 to 4.24 Mb, with GC contents ranging from 45.7 to 46.6 percent [51]. The sequencing results of the current study are similar to those of the previous investigation, in which the genome of *B. aryabhattai* (SQU-R12) isolated from date palm saplings was found to be 5.584 Mb in size and to have a GC content of 37.74% [52]. The chromosome sequence length of *B. aryabhattai* (SK1-7) isolated from the silver poplar rhizosphere was 5.188 Mb, with a GC content of 38.2% [53]. The chromosome sequence length of *B. aryabhattai* (AB211) isolated from the tea rhizosphere was 5.403 Mb, with a 37.8% GC content. The authors also summarized the genome sizes of various *B. aryabhattai* strains, which ranged from 5.0 to 5.7 Mb and had GC contents of 37.7 to 38.2% [54].

To understand the allelopathic mechanism of the *C. equisetifolia* endophytes XYG6 and XAG3 at the gene level, allelochemical synthesis-related genes and their relevant functional pathways were screened based on gene annotation information. The results of this study revealed that XYG6 and XAG3 share the allelochemical synthesis-related gene *aspB*, which synthesizes 4-hydroxyphenylpyruvate via the isoquinoline alkaloid biosynthetic pathway. 4-hydroxyphenylpyruvate dioxygenase (HPPD) catalyzes the conversion of 4-hydroxyphenylpyruvate to homogentistic acid (HGA), an important precursor for the biosynthesis of plastoquinone and tocopherol in plants. [55]. Previous research showed that plastoquinone is a biosynthetic precursor of carotenoid allelochemicals [56]. Similarly, naturally occurring polyketides can serve as allelochemicals by inhibiting HPPD and blocking the production of homogentisate and redox cofactors [57]. AspB and yhdR may synthesize L-phenylalanine and phenylpyruvate using tropane/piperidine and piperidine alkaloid biosynthesis, respectively. L-phenylalanine is a key precursor of phenolic acids and functions as a substrate in the synthesis of allelopathic phenolic acids [58]. The allelochemical indole can be synthesized by *trpA* through the phenylalanine, tyrosine, and tryptophan biosynthesis pathways [59]. Further research revealed the role of trpB in the conversion of indole to the allelochemical L-tryptophan, which has been demonstrated to be allelopathic in experiments involving wheat, oats, and rice [60]. Similarly, GGPS produces geranylgeranyl diphosphate (GGDP) via the terpene carbon skeleton biosynthesis route. GGDP is cyclized into momilactone allelochemicals, whose release can be stimulated by nearby plants and their root secretions to perform allelopathic effects [61]. Furthermore, the allelochemical synthesis-related genes NQO1, entC, rfbC/rmlC, adhP, and ghrB are unique to XYG6, and they can produce a variety of allelochemicals via secondary metabolite biosynthesis pathways. The allelochemical synthesis-related gene TYR, which is specific to XAG3, can produce 3,4-dihydroxy-L-phenylalanine (L-DOPA) and dopamine via the isoquinoline alkaloid route. L-DOPA is a precursor to many alkaloids, catecholamines, and melanin, which can be released into the soil and have a substantial allelopathic effect on surrounding plants [62]. This implies that B. amyloliquefaciens XYG6 and B. aryabhattai XAG3 may cause allelopathy by regulating the expression of genes associated with allelochemical synthesis in the biosynthetic pathways of secondary metabolites, terpenoids, ubiquinone, and other terpene quinones, as well as the biosynthesis of tropane/piperidine and piperidine alkaloids.

## 5. Conclusions

In conclusion, the whole genome sequencing of two potentially allelopathic Bacillus strains isolated from the roots of Casuarina equisetifolia provided useful insights into their genetic composition and potential ecological significance. We obtained genome-wide information on *B. amyloliquefaciens* XYG6 and *B. aryabhattai* XAG3 and screened their genomes for genes relevant to allelochemical production, as well as the biological processes relating to these genes. The *C. equisetifolia* endophytes *B. amyloliquefaciens* XYG6 and *B. aryabhattai* XAG3 share the genes aspB, yhdR, trpA, trpB, and GGPS, which are involved in the production of carotenoids, phenolic acids, indole, L-tryptophan, momilactones, and other allelochemicals. Furthermore, *B. amyloliquefaciens* XYG6 is involved in allelochemical synthesis, specifically the production of ubiquinone and other terpenoid quinones, as well as the polyketide unit biosynthesis pathway. *B. aryabhattai* XAG3 is involved in allelochemical synthesis largely via the terpene carbon skeleton biosynthesis pathway and the isoquinoline alkaloid biosynthesis pathway.

## Figures and Tables

**Table 1:** Characteristics of the XYG6 and XAG3 genome sequences.

Name	Genome Size (bp)	GC Content (%)	Plasmids	Coding Genes	tRNA	rRNA
XYG6	3854088	46.37	0	3744	86	27
XAG3	5507526	38.07	7	5580	134	42

## Data Availability

The data presented in this study have been deposited in the NCBI repository, accession number: PRJNA1021318, with the following URL: http://www.ncbi.nlm.nih.gov/bioproject/PRJNA1021318, accessed on 13 June 2024.
